# In-field determination of soil ion content using a handheld device and screen-printed solid-state ion-selective electrodes

**DOI:** 10.1371/journal.pone.0203862

**Published:** 2018-09-25

**Authors:** Ron Rosenberg, Michael S. Bono, Soumya Braganza, Chintan Vaishnav, Rohit Karnik, A. John Hart

**Affiliations:** 1 Department of Mechanical Engineering, Massachusetts of Institute of Technology, Cambridge, MA, United States of America; 2 Technology and Public Policy Program, Massachusetts Institute of Technology, Cambridge, MA, United States of America; 3 Sloan School of Management, Massachusetts Institute of Technology, Cambridge, MA, United States of America; Istituto Italiano di Tecnologia Center for Micro BioRobotics, ITALY

## Abstract

Small-holding farmers in the developing world suffer from sub-optimal crop yields because they lack a soil diagnostic system that is affordable, usable, and actionable. This paper details the fabrication and characterization of an integrated point-of-use soil-testing system, comprised of disposable ion-selective electrode strips and a handheld electrochemical reader. Together, the strips and reader transduce soil ion concentrations into to an alphanumeric output that can be communicated via text message to a central service provider offering immediate, customized fertilizer advisory. The solid-state ion-selective electrode (SS-ISE) strips employ a two-electrode design with screen-printable carbon nanotube ink serving as the electrical contacts for the working and reference electrodes. The working electrode comprises a plasticizer-free butyl acrylate ion-selective membrane (ISM), doped with an ion-selective ionophore and lipophilic salt. Meanwhile, the reference electrode includes a screen-printed silver-silver chloride ink and a polyvinyl-butyral membrane, which is doped with sodium chloride for stable reference potentials. As a proof of concept, potassium-selective electrodes are studied, given potassium’s essential role in plant growth and reproduction. The ISE-based system is reproducibly manufactured to yield a Nernstian response with a sub-micromolar detection limit (pK^+^ of 5.18 ± 0.08) and near-Nernstian sensitivity (61 mV/decade) in the presence of a 0.02 M strontium chloride extraction solution. Analysis of soil samples using the printed electrodes and reader yielded a correlation coefficient of 𝑅^2^ = 0.89 with respect to values measured via inductively coupled plasma atomic emission spectroscopy (ICP-AES). The reliable performance of this system is encouraging toward its deployment for soil nutrient management in resource-limited environments.

## Introduction

Increasing agricultural productivity is one of the most effective ways to alleviate poverty for the nearly 500 million small-holding farmers in the developing world [1,2]. For example, in agrarian economies such as India, it has been found that increases in agricultural productivity are 2.9 times more effective in improving rural poverty than any other equivalent gross domestic product (GDP) contribution [3]. And while there are many factors behind sub-par agricultural efficiency, including low mechanization [4] and lackluster pest management [5], poor soil health is one of the major contributors that can be targeted at the source. If more farmers were able to test their soil effectively and receive actionable advisory at the point of testing, they would be able to make more informed fertilization decisions, improve soil health, and increase economic output up to twofold [6].

In addition to impaired profitability for the farmer, improper fertilization can negatively affect the surrounding environment. Over-fertilization, for example, can cause severe downstream pollution, especially in nations such as India and China where it is common for nitrate and phosphate fertilizers to be applied at twice the requisite amount due to government subsidy [6]. Such practices ultimately lead to downstream water eutrophication [7], increased greenhouse gas emissions [8], soil acidification [9], and an overall decline in soil fertility. Soil diagnostic solutions, therefore, are critical players in maintaining a healthy and sustainable environment [1].

Technologies for analysis of soil ion content must measure concentrations of target ionic nutrients in the field and report these measurements in a clear and actionable manner. The most commonly measured target ions in soil tests are the macronutrients most critical to plant growth: nitrate (N), phosphate (P), and potassium (K). Micronutrients such as magnesium (Mg), copper (Cu), and zinc (Zn) are less salient to plant development, and thereby tested less frequently [10].

For both macro- and micronutrients, the prevalent soil test methods include atomic adsorption spectroscopy (AAS), colorimetry, flame photometry, and inductively coupled plasma atomic emission spectrometry (ICP-AES). The aforementioned methods generally provide high accuracy and detection limits well beneath the concentrations needed for actionable soil health assessment. However, with the exception of colorimetry, these techniques require trained laboratory personnel and expensive laboratory instrumentation (>1000 USD for flame photometry, >10,000 USD for AAS, and >50,000 USD for ICP-AES), necessitating analysis at centralized laboratory facilities such as agricultural extension centers [11]. Given that these centers are also typically mandated to provide a slew of other services, they generally lack the bandwidth to test all the farmers’ soil samples and provide fertilization recommendations in time for the upcoming growing season [11,12].

Colorimetric microfluidic devices have been considered as a potential method for decentralized soil chemistry analysis [13]. On the positive side, they utilize a simple transduction method (color), are low in cost, and can be implemented at the point of testing [14]. However, transitioning to mass-scale use has been impeded due to the need for multi-step assays with specialty equipment and environmentally-sensitive chemicals [15], as well as inaccuracy due to the inherent subjectivity of color interpretation. Our team’s user testing in India further underscored this sentiment, as farmers found colorimetric based tests from in field soil kits confusing and unreliable [16]. In order to achieve objective measurement, it is beneficial to use detection methods which can be easily transduced to an electric signal, which in turn facilitates direct numeric output without the need for interpretation. Electric transduction can be accomplished via simple conductivity measurements after sample separation via microchip capillary electrophoresis, enabling multiplex detection of soil macronutrients [17]. However, electrophoretic separation requires the use of high voltages (>1000 V) and more complex measurement protocols which limit feasibility for nutrient testing in resource-limited settings.

Ion-selective electrodes (ISEs) offer a simpler method for electronic transduction, which can be combined with low-cost electronic instrumentation for a promising point-of-use soil analysis solution without the drawbacks of colorimetry or capillary electrophoresis. Billions of ISE measurements are used annually for trace ion detection in physiological analysis, manufacturing process control, and environmental analysis [18,19], using a voltage-based measurement system that facilitates direct numeric output without the need for interpretation. ISEs can measure a vast array of ions, enabling multiplexed soil ion detection.

**Fig 1:**
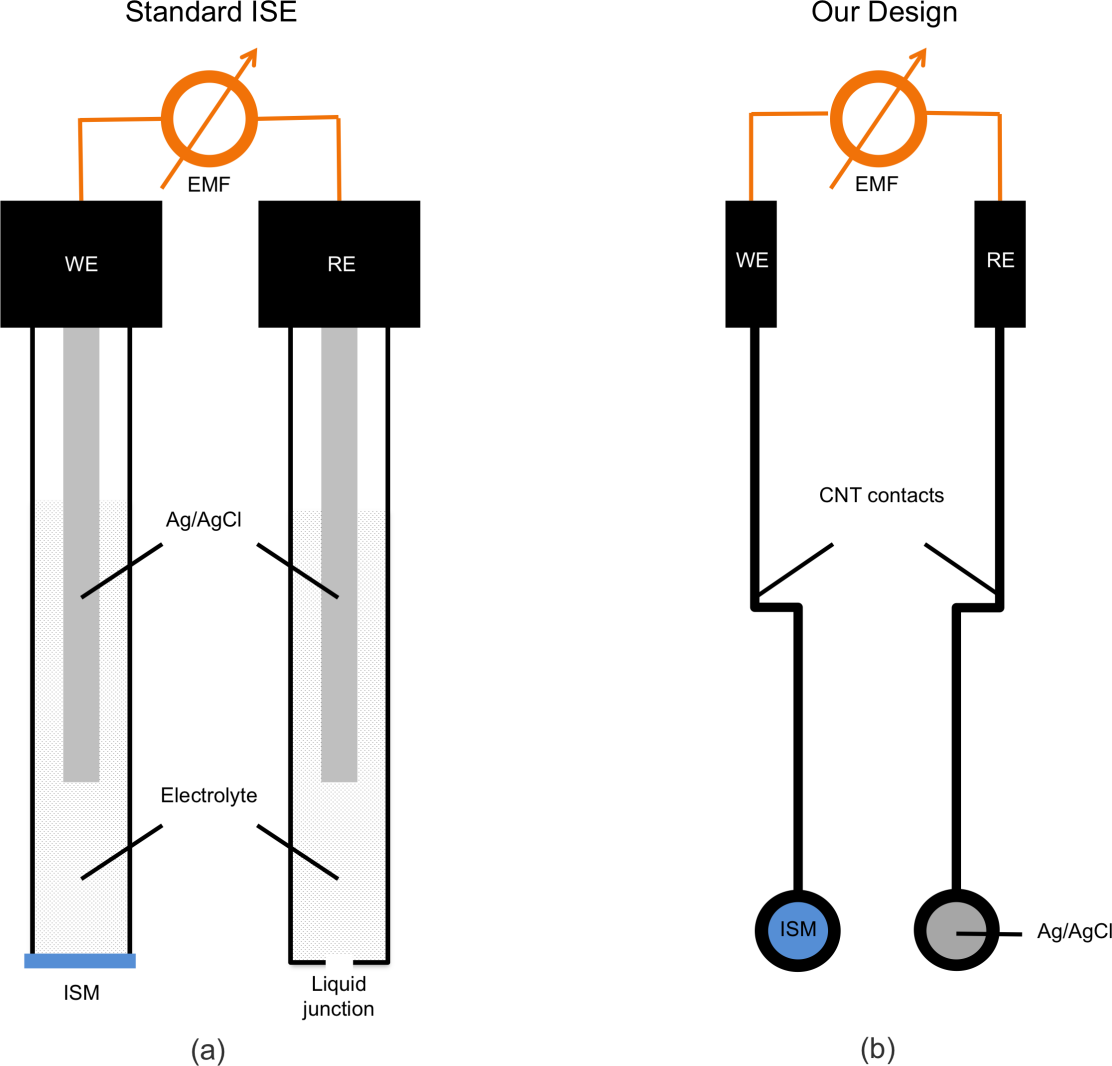
Schematic of (a) a typical liquid-phase double-junction ISE on the market juxtaposed with (b) a top-down view of our solid-state ISE.

ISEs are typically constructed as depicted in Fig 1a, with inputs from a working electrode and a reference electrode filled with electrolyte solutions. Within each electrode, a silver-silver chloride electrode (normally a chlorided silver wire) immersed in a known chloride ion concentration maintains a constant potential difference between the Ag/AgCl electrode and the corresponding electrolyte. The electrolyte in the reference electrode is connected to the sample solution via a liquid junction, which results in a liquid-junction potential difference that can be calculated via approaches such as the Henderson equation [20]. The liquid junction frequently contains high concentrations of equitransferent salts such as lithium acetate to minimize dependence of this liquid-junction potential on the sample analyte concentration [20]. The electrolyte in the working electrode is separated from the sample solution by an ion-selective membrane (ISM), which contains a carrier for the analyte ion, and the exchange of ions at the ISM/solution interface results in a potential difference that depends on the analyte concentration as described in the Results and Discussion section. The electrolyte in the working electrode contains the analyte ion at a known concentration, which results in a constant potential difference at the electrolyte/ISM interface. Thus, for a correctly designed ISE, the only potential drop which depends on the analyte concentration will be the potential difference at the ISM/solution interface, and the ISE can then be used for potentiometric measurement of analyte ion concentration.

Despite their commercial success and wide application, ISEs remain mostly inaccessible outside of laboratory settings. A major reason for this is that the measurement configuration of conventional ISEs results in high sensor costs upwards of thousands of dollars (including the reference electrode), large footprints (e.g., >100 mm in length), as well as the requisite attention to avoid evaporation and crystallization of the electrolyte [21]. These difficulties can be mitigated through the use of miniaturized, solid-state ion-selective electrodes (SS-ISEs) that measure the potential within the membrane directly without the use of a filling solution, where the working potential for SS-ISEs is described in the Results and Discussion Section.

State of the art SS-ISEs have come a long way since the first coated wire SS-ISE was proposed by Hirata and Date in 1970 [22]. In this configuration, a platinum wire serving as the electrical contact was coated with a copper-sulfide impregnated silicone rubber that complexed copper ions. While their electrode was revolutionary in its approach, a major drawback was the thermodynamically ill-defined interface at the wire-membrane interface, where ionic charge at the membrane surface must be electrochemically converted to electronic charge at the metallic conductor surface for a potential measurement to occur. The absence of a redox couple between the electronically conducting wire and ionically conducting membrane leads to unstable potential measurements. Further, as reported in 2000 by Pretsch et al [23], this type of ISEs had the additional problem of an unintentional water layer formed at the contact-membrane interface, leading to potential drifts, unfavorable detection limits, and delamination of the sensing membrane from the electrical support [24].

In order to circumvent the aforementioned problems, the use of a transducer layer was introduced [24–27]. This requires a solid material capable of converting ionic charge to electronic charge, whether via a redox reaction at the transducer-membrane interface (i.e., redox capacitance) or via charge generation in the electric double layer at the transducer-membrane interface (i.e., electric double layer capacitance) [24]. Reliable transducer layer performance also requires superhydrophobicity to avoid water layer formation. A water layer is deleterious to ISE performance as even small ionic impurities can have a considerable effect on the membrane-conductor phase boundary potential [28]. The first ion-to-electron transducers suggested were conjugated polymers such as polyaniline, polypyrrole, and PEDOT-PSS, all of which function via redox capacitance [24] and were successful in improving potential stability and reducing detection limits [29].

While conducting polymers offer the advantages of high redox capacitance, low cost, and ease of processing, they suffer in terms of their light sensitivity and insufficient hydrophobicity, which leads to water layer formation. New directions in solid-state ISEs have focused on incorporating high specific area carbonaceous materials, which have shown to offer high electric double layer capacitance and superhydrophobicity [28]. In one such configuration, carboxylated single walled carbon nanotubes (CNTs) were used as a transducer layer for potentiometric strip cells based for potassium measurements [30]. CNTs are particularly well-suited for use as transducer layers due to their chemical inertness, hydrophobicity, high surface area, and surface electron mobility, resulting in a highly sensitive electronic response to the potential generated by the membrane ions in the electric double layer at the membrane-transducer layer interface. This compares favorably to the redox capacitive response of conducting polymers while decreasing the likelihood of water layer formation [31,32].

Typically, the transducer layer is constructed above the electrode material or above a noble metal contact. With gold and platinum noble contacts, sub-micro molar limits of detection have been realized even for potassium ISEs [33]. For instance, a recent study used CNT-painted conductive paper with a conductive polymer functionalized gold solid contact and a poly(methyl methacrylate)–poly(decyl methacrylate) sensing membrane to achieve nanomolar detection limits for Cd^2+^, Ag^+^, and K^+^ [34]. However, the detection limit improvement came at the expense of increased manufacturing complexity, including sputtering of the gold, multi-step painting of the paper, and drop casting of the conductive polymer layer.

As such, there is considerable interest in simplifying the SS-ISE architecture, manufacturing process, and material cost. In one example, Michalska et al. reported a disposable ISE, with CNT-doped PVC layer serving the dual purpose of the electrical contact and ion-to-electron transducer and PEDOT as the transducer layer [35]. However, as mentioned above, conductive polymer transducer layers suffer from drift and light sensitivity. Meanwhile, Whitesides and colleagues recently demonstrated a low-cost potentiometric cell for potassium measurement based on wax-modified filter paper and screen-printed Ag/AgCl inks, though a relatively high detection limit of 0.1 mM was found [36]. Beyond manufacturability, previously implemented SS-ISEs have not addressed the challenges associated with determining ion content in complex soil-extract matrices. The ionic interferences presented by soil testing make it more challenging to achieve high membrane selectivity and detection limits. Additionally, soil samples have to be treated with soil extraction solutions, which aid the removal of ions from the soil colloid complex through the use of concentrated salts. Selecting a universal extraction solution which does not interfere with SS-ISE performance is therefore important to achieve multiplexed soil ion detection.

In addition, few examples in SS-ISE literature target the need for a low-cost, portable potentiostat for on-site soil testing. Devices such as the CheapStat and DStat are successful advances for sub-100 dollar open-source electrochemical readers [37,38], but they contain excessive features relative to what is needed for simple open-circuit potentiostatic measurements. Further cost reduction can be achieved with a simpler reader tailored to SS-ISE operation in the field.

**Fig 2:**
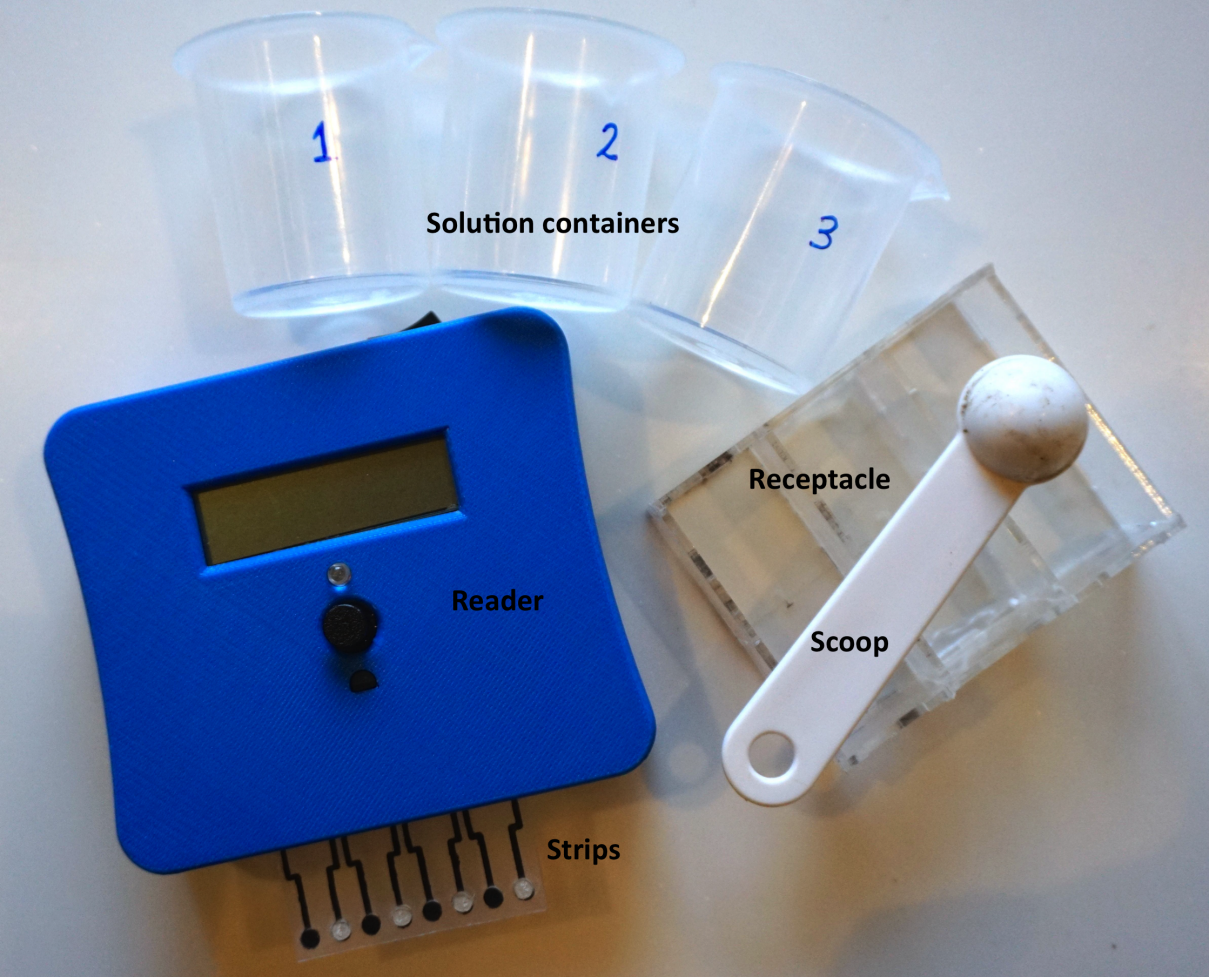
The complete ISE system. System consists of (i) the solution containers 1, 2, 3 for the extraction solution, deionized water wash, and calibration solution; (ii) the three-compartment receptacle to hold the solutions and soil; (iii) the scoop to scoop the soil sample; (iv) the ISE strips; and (v) the handheld electrochemical reader.

This paper details the fabrication and characterization of an integrated point-of-use soil testing system, including a set of disposable SS-ISE strips and a handheld electrochemical reader (Fig 2), which together meet the aforementioned challenges of cost, manufacturing complexity, soil interferences, and on-site testing. In the following, we present (1) a fabrication method for paper-based SS-ISEs using potassium-selective ISEs as a proof of concept; (2) demonstration of sensor sensitivity and reproducibility of ± 3.24 mV/decade, which are critical metrics for accurate soil test results; (3) verification of low (sub-mV) interferences from competing soil ions; (4) validation of strontium chloride as a universal extraction solution which maintains ISE sensitivity and detection limits in presence of interfering ions; and (5) the integration of the ISEs in a prototype field test kit including a handheld electrochemical reader which performs measurements using the printed ISEs and converts them into an alphanumeric output for field use.

## Materials and methods

### Sensor architecture

**Fig 3:**
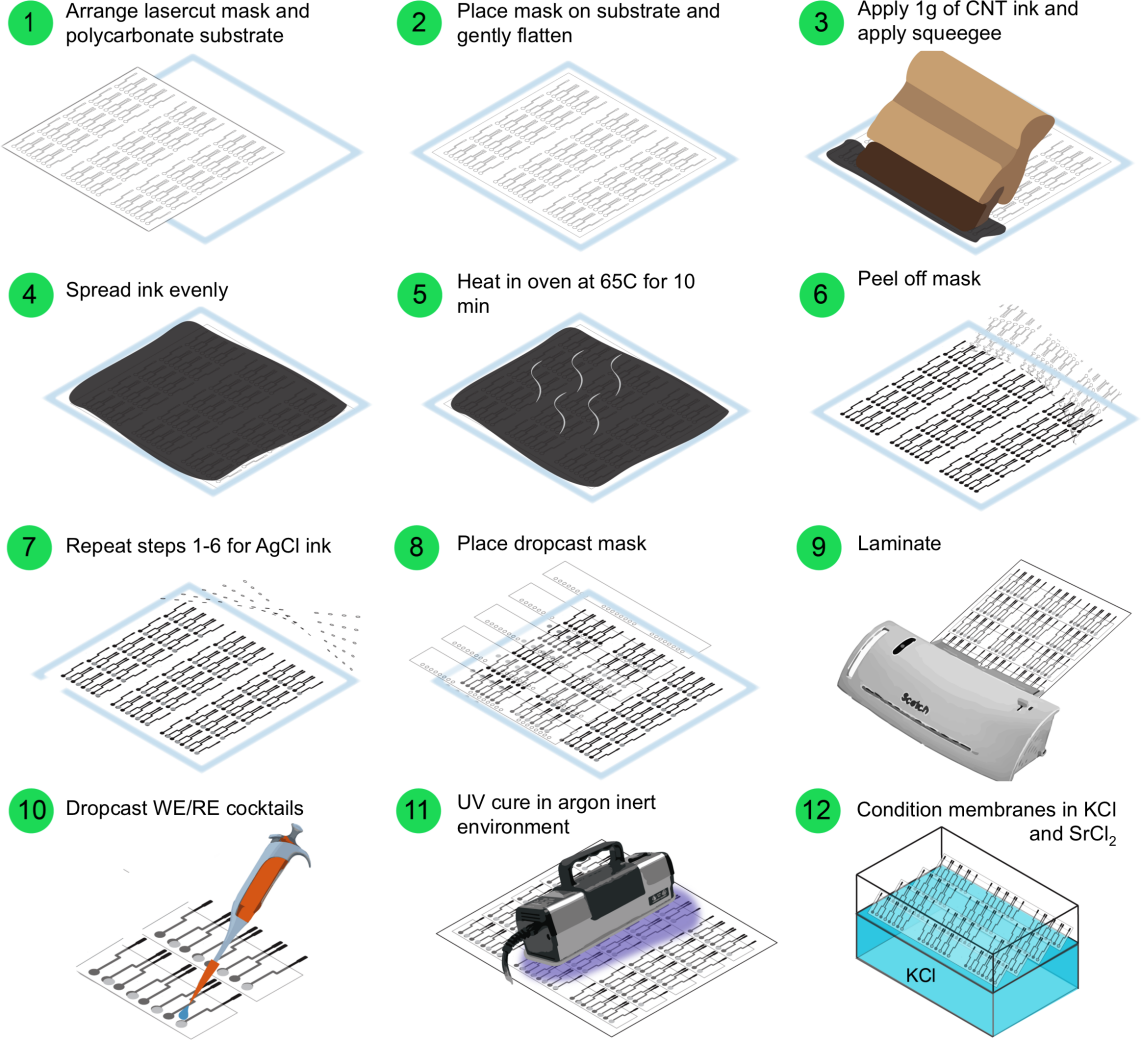
A step-by-step schematic of the manufacturing process. Manufacturing process begins with laser cutting masks for screen printing and ends with conditioning of the ISE strips in standard solution.

The printed ISE sensor architecture is shown in Fig 1b and a step-by-step schematic of the manufacturing process is shown in Fig 3. On a single sheet (8.5x11”), 96 individual electrodes (i.e., 24 strips of four sensors each) were produced. Screen-printing masks were made of Grafix paper (Maple Heights, OH, USA) and the ISM dropcasting mask was made of 0.005” thick ultra high molecular weight polyethylene (UHMWPE) with adhesive backing (McMaster-Carr, Elmhurst, IL, USA). All masks were cut using a 60 W CO2 laser cutter (Epilog, Golden, CO, USA).

The working and reference electrodes were 1.25 mm (W) x 28 mm (H). At the top of the electrodes the width increased to 1.59 mm, and the spacing of 7.5 mm apart was chosen to accommodate the required edge card reader spacing inside of the printed circuit board of the handheld reader (S1 Fig). Two circular holes of 4.5 mm diameter for the CNT ink were centered on the bottom end of the working and reference electrodes were. The diameters of the Ag/AgCl layer and the dropcast mask diameter were reduced to 4 mm and 3.5 mm respectively so as to accommodate for alignment accuracy of the masks (placed manually) and to ensure that the Ag/AgCl layer or ISM were always in contact with the CNT layer beneath.

### Electrode fabrication

To fabricate the electrodes, the bottom layer was first screen-printed using 7102 CNT conductor paste from DuPont (Wilmington, DE, USA). A laser-cut (0.003-inch thickness) Grafix film masker was carefully peeled off from the adhesive back, and placed onto a 0.015-inch-thick polycarbonate substrate purchased from McMaster and cut to a standard 8.5 by 11 inch size. 1 g of the 7102 CNT paste was spread on top of the mask and manually spread across with the help of an 80-durometer polyurethane squeegee from Speedball (Statesville, NC, USA). The ink was cured in the oven at 70°C for 10 min and the CNT mask was then peeled off and disposed. The same process was followed for printing the Ag/AgCl layer using 5874 Ag/AgCl paste from DuPont, with the exception of a reduced heating time of 5 min so that the acrylic adhesive of the mask would not stick to the CNT layer beneath. With the CNT and Ag/AgCl layers complete, single row laser-cut strips of the UHMWPE was carefully aligned and applied by hand. The masked polycarbonate was then placed in a plastic lamination machine (Scotch Brand, 3M, Minneapolis, MN, USA) to ensure a complete seal between the acrylic adhesive and the substrate.

### Membrane fabrication

The ion-selective membrane layers were formed by dropcasting the mixtures for the reference and working electrode membranes. The mixture for the reference electrode (RE) membrane consisted of 79.1 mg polyvinyl butyral (PVB) and 50 mg sodium chloride (NaCl) dissolved in 1 mL methanol; all were reagents purchased from Sigma-Aldrich (St. Louis, MO, USA). The mixture was sonicated at 75 Hz in a sonicator bath (Branson Model 2510, Branson Ultrasonics, Danbury, CT, USA). Next, 10 μL of the mixture was dropcasted using a positive displacement pipette (VWR) and left to dry at ambient conditions for 12 hours.

To formulate the working electrode (WE) membrane, 12 mg valinomycin, 3 mg potassium-tetrakis 4-chlorophenyl borate (KTpClB), 3 mg tetrakis(4-chlorophenyl)borate tetradodecylammonium salt (ETH500), and 7 mg 2,2-dimethoxy-2-phenylacetophenone (DMPP) were dissolved in a stock mixture consisting of 500 μL butyl acrylate (BA) and 2 μL hexane-1,6 diol diacrylate (HDDA).

After the RE membranes were dry, the strips were moved to a glovebox (with argon atmosphere) and 2 μL of the WE membrane cocktail was dropcasted through the mask using a manual repeating pipette (USA Scientific, Ocala, FL, USA). The devices were then cured in the glovebox for 8 minutes under 365-nm light using two Spectroline 5W E-series lamps (Spectronics Corporation, Westbury, NY, USA).

After curing, the strips were removed from the glovebox and left in ambient conditions overnight before conditioning in 10^−3^ M potassium chloride (KCl) for 12 hours and then 3 hours in 2x10^−2^ M strontium chloride (SrCl_2_). Electrodes were conditioned in strontium chloride in order to saturate the PVB membrane on the reference electrode with chloride ion. Otherwise, when the ISEs would later be exposed to the strontium chloride extraction solution, there would be a net flux of chloride ion into the reference membrane from the extraction solution, which would shift the reference potential established by the chloride redox reaction at the Ag/AgCl layer. After conditioning, the strips were rinsed with deionized water (18.2 MΩ) and left in dry storage prior to use. No further preconditioning was necessary prior to electrochemical measurements.

### Physical characterization

Surface profiles were measured using a Dektak XT Profilometer (Bruker, Billerica, MA, USA). Average height and surface roughness were calculated by applying a linear fit filter. Scanning electron microscope (SEM) images were taken using a Zeiss Merlin Gemini SEM (Zeiss, Oberkochen, Germany). Prior to SEM imaging, ISEs were sliced with a razor blade to expose a cross section co-linear with the center of the ISM, and gold (5 nm thickness) was sputtered onto the electrodes at a vacuum pressure of 10^−6^ Torr to aid SEM imaging.

### Electrochemical characterization

Potentiometric measurements were taken using an 8-channel high impedance potentiostat (Solartron Model 1470E, AMETEK Scientific Instruments, Berwyn, PA, USA) at room temperature using a double-junction setup with two beakers (one each for the working and reference electrodes) connected by a lithium acetate salt bridge. Due to the zero-current nature of potentiometric measurement, no counter electrode was necessary for accurate potential measurement. For independent measurements of the PVB-NaCl REs or BA-ISM WEs, a commercial reference electrode purchased from Gamry (Warminster, PA, USA), consisting of Ag/AgCl in 3 M KCl, was immersed in a 10^−2^ M KCl solution, and connected to the WE sample bath via a 1.5 cm by 20 cm filter paper (Whatman) salt bridge hydrated with 200 μL of 1 M Lithium Acetate (LiOAc). Concentrations of the primary ion were varied by adding fixed amounts of different molarity stock solutions to achieve decade increases in concentrations of the sample bath. Measurements of the full ISE system, including the PVB-NaCl RE and BA-ISM WE, were done in a single sample bath, with similarly increasing concentrations of primary ion. All potentiometric values were corrected for the liquid-junction potentials according to the Henderson equation [20] and the ion activities were calculated by the Debye-Hückel equation [20,39].

Selectivity measurements were completed via the fixed interference method (FIM) [40] with the fixed activity of all interfering ions at 1 mM, a level representative of typical soil analyte concentrations. 𝐿𝑜𝑔𝐾_𝑖𝑗_ selectivity constants were calculated for all interfering ions as the activity corresponding to the intersection of the potentiometric titration curve with and without the interfering ion present. Required selectivity coefficients were calculated using the respective typical concentration of the interfering ion in soil, and an upper bound percent error of 5%.

A Python script was written for data analysis. The Python script sorted the acquired values into a 10-bin histogram, after which a normal Gaussian distribution was fit to the data. The standard deviation and mean were reported to assess the reproducibility.

### Soil sampling and analysis

Samples from 30 locations around the MIT campus in Cambridge, MA, USA (42.36 N, 71.09 W) were collected at 20-cm depth with a soil core. For each respective sample, 5 sub-samples were combined and mixed into one to provide a representative sample. The samples were air dried and sieved to less than 2 mm after crushing aggregates manually. Particles greater than 2 mm (e.g., pebbles and stones) were discarded.

For each soil analysis, two grams of air-dried soil was placed in a 50 mL vial and extracted with 20 mL of either 0.02 M strontium chloride, Kelowna extract (0.25 M CH_3_COOH + 0.015 M NH_4_F), Morgan extract (1.4 M NH_4_OAC+1 M HCl + 0.025 M EDTA), or modified Morgan extract (0.62 M NH_4_OH + 1.25 M CH3COOH) (1:10 soil-weight: solution-volume). Vials were placed on a rotary mixer for 15 min at 180 oscillations per minute (opm). Filtrates were collected using a Whatman 4 filter paper and diluted at a 10/3 ratio with a 2% Nitric Acid solution. Potassium standards with concentrations of 1, 10, and 100 ppm were prepared from KCl (Sigma) and matrix-matched and diluted equivalently with the same Nitric Acid solution. Standards and samples were measured on an Agilent (Santa Clara, CA, USA) 5100 ICP-AES and calculated via a three-point linear regression.

For soil characterization via the fully integrated ISE system, soil samples were diluted 20x (for sufficient solution volume) and characterized using a one-point calibration against a standard solution of 1 mM KCl in a background of 0.02 M strontium chloride. Calibration and sample values were recorded for each step outlined in the supporting information (S1 Protocol) and transduced to soil concentrations by the Nernst equation and the necessary conversion factors.

## Results and discussion

### Ion-selective electrode fabrication by screen printing

The SS-ISE sensors were designed as a two-electrode system [41] fabricated on plastic substrates (Fig 1b) and integrated with a custom-built handheld reader for soil ion measurement (Fig 2). The device architecture was simplified by utilizing the single-walled CNT layer as both the ion-to-electron transducer and electrical contact.

The CNTs were screen-printed onto the polycarbonate substrate, eliminating the need for expensive deposition equipment and requiring only a laser-cut mask and a squeegee. The heat-laminated UHMWPE mask layer simultaneously protected the electrodes from abrasion and water intrusion, insulated the potential signal from stray currents, and constrained the drop-casted membranes to the desired regions.

**Fig 4:**
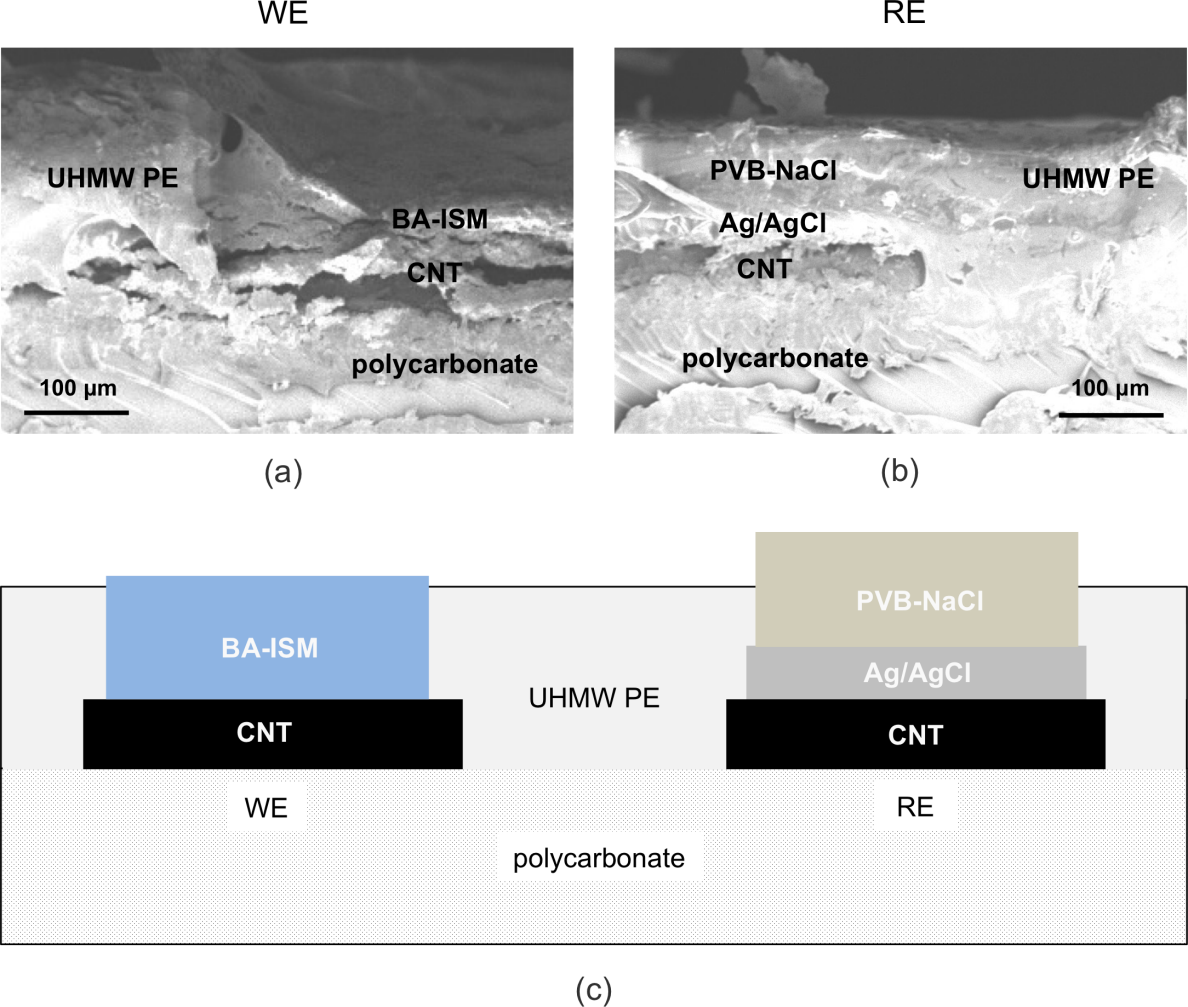
Cross sectional SEMs of the WE (a) and RE (b) located at the aperture of the respective electrodes to the solution, and (c) a schematic of the same location.

Physical characterization of the fabricated SS-ISEs demonstrated that this process yielded repeatable device morphology, which in turn gave highly reproducible potentiometric responses. Scanning electron microscopy (SEM) imaging of the completed electrodes showed excellent adhesion among the layers (Fig 4), and profilometry of the individual layers showed repeatable layer thicknesses from our described fabrication protocol. For the electrode contacts, the CNT and Ag/AgCl layers exhibited rough morphologies and thicknesses of 31 ± 3 μm and 39 ± 5 μm, respectively, with differences between the layer thicknesses likely due to the higher mass fraction of solid in the AgCl ink post-evaporation.

The drop-casted butyl-acrylate (BA) ISMs showed a smooth morphology, with thickness reproducibility of 127 ± 12 μm. Surface morphology and consistency were very sensitive to oxygen or water impurities in the air, so it was essential to fabricate the ISMs in a glove box environment. While the PVB/NaCl RE membranes were rougher than the ISMs, they demonstrated comparable thickness reproducibility (138 ± 14 μm) to the ISMs. Use of a positive-displacement pipette to fabricate the reference electrode membrane was essential to uniformity as it ensured minimal solvent evaporation or bubble entrapment during the drop casting process.

### Potentiometric response of screen-printed ISEs

The SS-ISE is constructed such that the ISM and Ag/AgCl are placed directly on carbon electrodes (Fig 4). Ideally, the potential drops at the carbon/ISM and carbon/Ag/AgCl interfaces are stable and constant. In place of the electrolyte and liquid junction in a regular liquid-filled ISE, the Ag/AgCl electrode is coated with a solid phase with a fixed chloride concentration that determines the potential at the Ag/AgCl/material interface. This solid phase is designed to be non-selective, so that the potential across the material/sample interface is independent of sample composition. Therefore, the key sensing mechanism of the printed ISEs occurs at ISM/sample interface of the working electrode, via the phase boundary potential (PBP) between the butyl-acrylate ISM and the aqueous sample. The PBP at the membrane-solution interface arises due to potassium in solution selectively diffusing between the aqueous phase and the membrane, doped with a KTpClB lipophilic salt and ion-selective carrier^,^ valinomycin [42].

While the resulting potential difference yields nanoscale electric double layers on both sides of the membrane-solution interface, in situations where the potential difference is due solely to the selective transport of potassium, the total potential difference can be calculated by solving for electrochemical equilibrium between the two phases. This potential is described by the Nernst equation,

$$E=E_{0}+\frac{RT}{z_{i}F}\ln a_{i}.$$

Here, 𝐸 is the measured potential, *E*_*0*_ is a constant potential, 𝑅 is the universal gas constant, 𝑇 is the absolute temperature, *z*_*i*_ is the charge on ion *i*, 𝐹 is the Faraday constant, and *a*_*i*_ is the activity of ion *i*.

An ideal Nernstian response in this case is characterized by a sensitivity (corresponding to the slope of a calibration curve) of 59 mV/decade for monovalent cations such as potassium at room temperature. This response, however, is predicated upon the activity of the primary ion in the bulk membrane phase being constant and sample-independent [41]. Any sample dependence would yield non-Nernstian behavior.

When characterized in deionized water, the fabricated SS-ISEs (N = 20) exhibited sub-Nernstian responses with a sensitivity (corresponding to the slope of the calibration curve) of 53.29 ± 2.78 mV per decade potassium concentration for *pK*^+^ = −log_10_[*K*^+^] greater than the mean detection limit of 6.22 ± 0.48. The measured mean detection limit corresponds to 24 ppb K^+^, well below typical minimal soil K^+^ levels of between 1 and 60 ppm [29].

However, actual field analysis of soil samples requires robust performance in the presence of interfering ions. With non-ideal selectivity of the membrane, the presence of such impurities can reduce the limit of detection, especially in the background of extraction solution with high molarity cationic interferences. As a result, validation of membrane selectivity along with an extraction solution that gave a satisfactory detection limit was a key criterion in development of an effective field-based soil analysis system. The phase boundary potential of a membrane in contact with an aqueous phase containing both the analyte of interest and interfering ionic species is given by the Nikolski equation,

$$E=E_{0}+\frac{RT}{z_{i}F}\ln\left(a_{i}+K_{ij}a_{j}^{\frac{z_{i}}{z_{j}}}\right).$$

Here, *K_ij_* is the selectivity coefficient of an interfering ion *j* with charge *z*_*j*_. Notably, for a perfectly selective membrane, the Nikolski equation reduces to the Nernst equation.

To test cross-sensitivity of the electrodes, a fixed interference method (FIM) approach [40] was used and the Nikolski coefficients *log K*_*ij*_ were reported. The resulting selectivity coefficients (Table 1) show that for nearly all interfering ions of interest, the BA ISMs demonstrate sufficient selectivity towards potassium so as not induce a significant error in the potentiometric measurement. Required selectivity coefficients were determined on the basis of introducing a 5% error, using typical concentrations of the interfering ion in New England soils [43]. The only ion with a potential for significant interference was ammonia, as it is highly concentrated in many soil fertilizers.

**Table 1:** Selectivity coefficients calculated using the fixed interference method (FIM). Required values were based on typical concentrations of respective analytes in New England soils [43] and a corresponding 5% error in potential measurement induced by the interfering analyte.

Ion	LogK_ij_	Required
Na+	-2.6 ± 0.10	-1.0
Mg^2+^	-4.3 ± 0.15	-1.8
Ca^2+^	-4.7 ± 0.28	-2.0
Li^+^	-2.3 ± 0.21	-1.3
NH_4_^+^	-1.7 ± 0.02	-1.0

**Fig 5:**
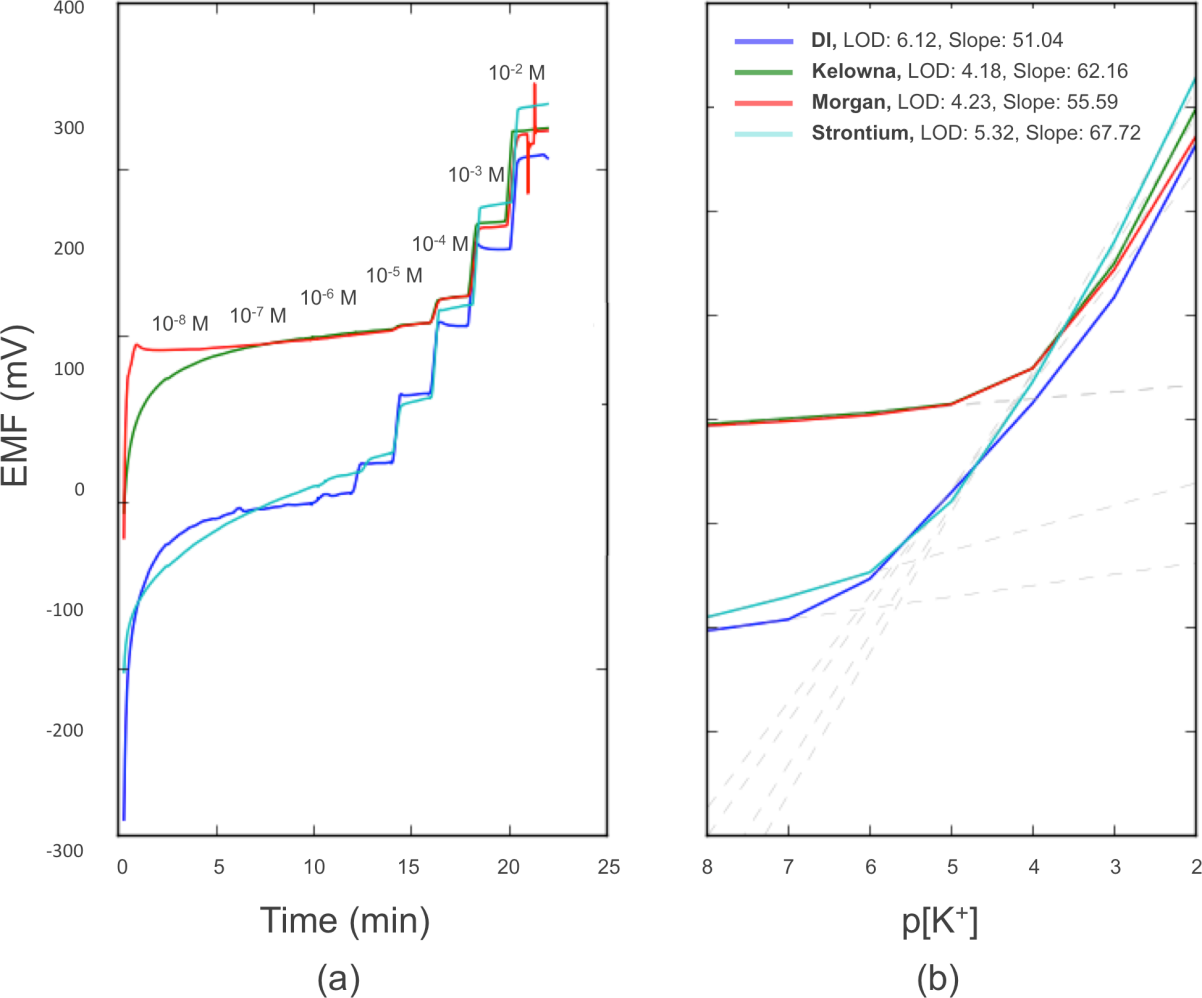
Qualification of the effect of various extraction solutions on the full SS-ISE system potentiometric response with deionized water background as the control. Response is shown as (a) potential versus time, and (b) potential versus logarithm of potassium activity.

Next, the response of the WEs in a background of a commonly used extraction solutions was tested in a broad concentration range from 10^−8^ to 10^−2^ M of aqueous KCl. As shown in Fig 5, 0.02 M strontium chloride outperformed the other extraction solutions by maintaining low detection limits. Strontium chloride degraded the detection limit of the WEs by less than one order of magnitude relative to the DI control, whereas Modified Morgan and Morgan UES degraded detection limits by nearly two orders of magnitude. Deterioration in performance of the Modified Morgan and Morgan solutions was likely due to their high molarity of ammonia, which according to Table 1 showed the greatest interference. While strontium was not tested as an interfering ion, the relatively low degradation in detection limit when interacting with such high strontium concentrations suggests that strontium poses a limited interference risk.

An interesting side effect of testing the WEs in the presence of strontium chloride was their relatively super-Nernstian response. In DI water, the samples tested exhibited a sub-Nernstian sensitivity of 53.29 ± 2.78 mV/decade as mentioned previously, while the sensitivity increased to 67.72 mV/decade for the strontium chloride background samples. It is likely that the respective sub-Nernstian and super-Nernstian responses of the samples in DI water and strontium chloride backgrounds can be attributed to the chloride ion concentration of the background. In the case of strontium chloride, the high molarity of chloride ion on the aqueous side of the interface likely promoted non-stoichiometric (that is, greater than 1:1) complexing of potassium with the carrier in the membrane phase [44]. The opposite would be true in the case of DI water. Notably, as seen later, the super-Nernstian response of the WEs in the strontium chloride background was moderated when testing the full ISEs.

For the reference electrode, we observed that the strontium chloride extraction solution has the ability to stabilize potentials in the presence of interfering ions. This is likely due to the high molarity of chloride ions in the extraction solution, which saturate the PVB membrane and then undergo a redox reaction at the Ag/AgCl layer. The saturation of the chloride ions around the Ag/AgCl layer makes this redox reaction less susceptible to minor changes in chloride concentration and thereby more stable in the presence of interfering ions.

**Fig 6:**
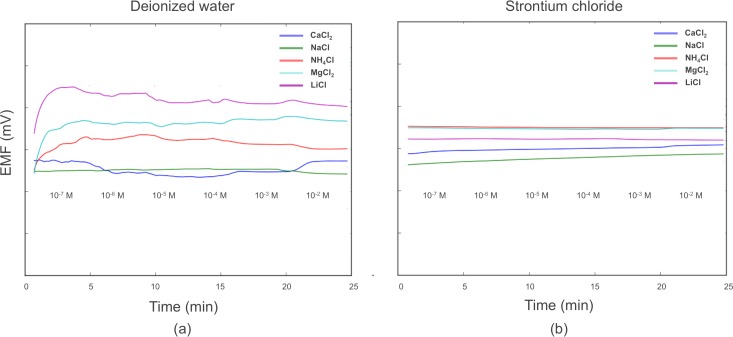
**Comparison of RE stability over potassium concentration ranges with (a) deionized water as the background and (b) 0.02 M strontium chloride as the background.** In this case, the SS-ISE RE was used as the working electrode and a Gamry 3 M KCl Ag/AgCl reference electrode was used as the reference.

In Fig 6, we show that, regardless of interfering salt concentration, a background of strontium chloride stabilizes reference electrode potentials. From one concentration range to another, potential shifts in the strontium chloride case were barely measurable, suggesting drift as the major contributor for the shift over the whole concentration range. Even with this drift, REs in lithium chloride and magnesium chloride varied only varied ten to hundreds of microvolts, a testament to the stabilizing force of strontium chloride on the reference electrode.

**Fig 7:**
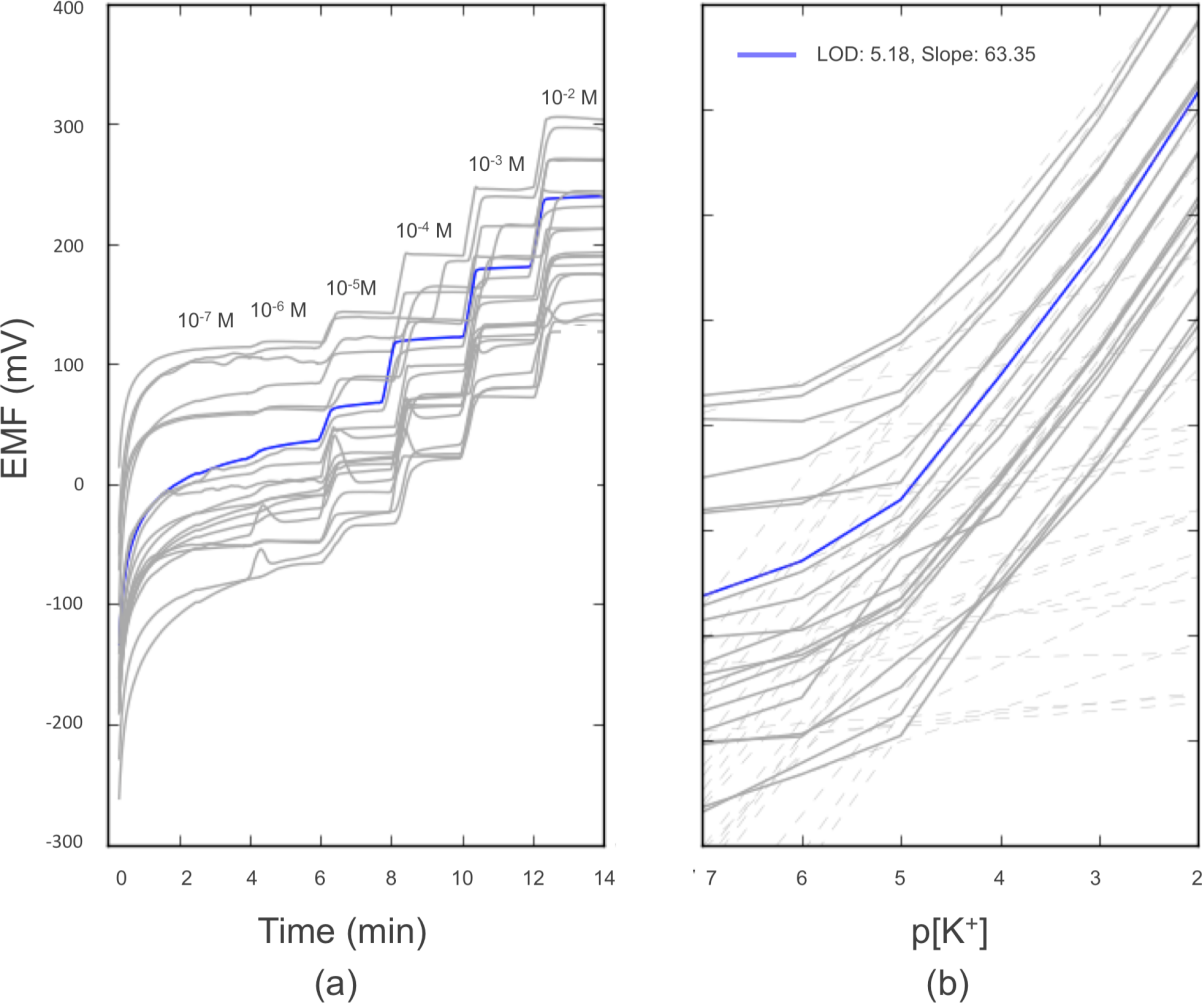
Reproducibility plot (N = 20) of the full ISE system response (WE versus RE) in the background of 0.02 M strontium chloride extraction solution using the external Solartron potentiostat. Response is shown as (a) potential versus time, and (b) potential versus logarithm of potassium activity. For readability, one representative response is shown in blue and the other replicates are shown in gray.

As seen in Fig 7, the full ISE system (WE and RE) exhibited highly reproducible potentiometric performance for measuring potassium in the presence of the strontium chloride extraction solution, with a sensitivity of 61.5 ± 3.24 mV per decade potassium concentration for pK^+^ greater than the mean detection limit of 5.18 ± 0.08 (N = 20), corresponding to 260 ppb K^+^ which is still less than typical soil potassium contents. It should be noted that the vertical dispersion of the curves in Fig 7 does not affect reproducibility, as the one-point calibration procedure (as described in the Materials and Methods section) removes absolute potential as a source of variability and renders membrane sensitivity as the key limiting factor in measurement reproducibility. As described above, this performance is more than adequate for determining potassium concentration in the range found in soil. Therefore, using our process, an ISM-based measurement system for potassium determination can be easily fabricated with only 5.3% variation in sensitivity and only 1.6% relative variation in limit of detection. Given that the sensitivity of the potassium response had a standard deviation of 3.24 mV/decade and soil K^+^ values span approximately two decades of concentration [29], this would suggest that the full ISE system (WE and RE) variability would constitute a total standard error of 6.28 mV in potential measurement.

#### Electrochemical reader for an integrated point-of-use system

Implementation of the printed ISEs for in-field soil analysis also requires a portable means of electrochemical measurement and a means of converting the measurement into actionable recommendation for the user. Using mobile-phone based healthcare as inspiration, we designed an electrochemical reader to convert soil ion content into an alphanumeric output, which could be connected to a central service provider–either directly using an embedded mobile phone chipset or indirectly by the farmer text messaging the measurement code to the system on their mobile phone–to give immediate fertilizer recommendations. By leveraging the mobile phone, which has seen impressive penetration across the developing world in the last decade [45], the reader can be kept as simple as possible.

A detailed description of the reader circuitry can be found in the supporting information (S1 Text). Briefly, the reader board was designed with two stages of operational amplifiers. The first stage served as a buffer to minimize stray current going into the working electrode and thus ensure the zero-current condition for potentiometric measurement. The second staged served as an amplifier to make signal readable by the microprocessor. In addition, we fabricated a 3D-printed case (S3 Fig) and a laser-cut acrylic receptacle to facilitate on-site testing, including ion extraction, wash, and calibration.

**Fig 8:**
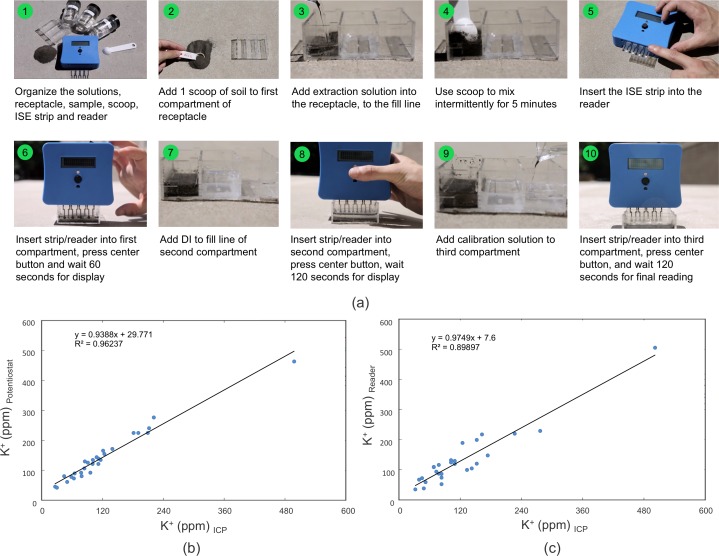
**(a) Step-by-step instructions for how to take an ISE measurement using the SS-ISE strips and the handheld reader (b,c) Estimated potassium concentration of 28 soil samples as measured via potentiometry of a single SS-ISE strip.** Estimated concentration is plotted with respect to concentration as measured via ICP-AES, where the potentiometric response is measured using (b) a commercial Solartron potentiostat and (c) the manufactured reader.

The procedure of soil testing with the in-field kit (reader, strip, and receptacle) is shown in Fig 8. The procedure is as follows. First, the farmer takes a composite soil sample on the farm. Then, he/she adds an aliquot of the soil sample and extraction solution into the first receptacle and stirs for 5 minutes. The disposable ISE strip is inserted into the reader and then both are inserted into the first receptacle for measurement. After a wash with deionized water and subsequent calibration step with a standard solution, the reader deciphers the soil potassium content into a numerically binned output of “1”, “2”, “3”, “4”, or “5”; representing “Low” (1–50 ppm K^+^), “Medium-Low” (51–150 ppm K^+^), “Medium” (151–250 ppm K^+^), “High” (250–800 ppm K^+^), or “Excessive” (> 800 ppm K^+^). For our field trials in India, the back of the reader contained a card with letters “A”-”Z” associated with crop translations in the local language. Users could therefore add these letters at the end of the numeric part of the text to receive customized feedback for a particular set of crops.

The total predicted system error (S2 Fig) for the SS-ISEs and the portable reader together using an RSS summation of individual errors yields approximately 6.35 mV. Such a potential error would yield a 74% accurate bin prediction rate (i.e. “Low”, “Medium”, “High’) according to an error model further described in the supporting information (S2 Fig).

### Soil sample analysis using the integrated system

To evaluate the performance of the point-of-use device in comparison with conventional laboratory-based soil testing, samples were measured both with the ISEs and the ICP. In Fig 8, we show correlation of calculated potassium concentrations for 28 soil samples measured using fabricated ISEs interfaced with either a potentiostat or the fabricated electrochemical reader. All 30 measurements (of which 2 were rejected due to improper handling) were taken using the same ISE and a one-point calibration of the sample compared to 10^−2^ M KCl in a background of 0.02 M strontium chloride, waiting 90 seconds after insertion of ISE into solution to avoid transient responses. ISE responses measured on the potentiostat exhibited a correlation of 𝑅^2^ = 0.96 with respect to ICP-AES samples, while those measured on the portable reader exhibited a correlation of 𝑅^2^ = 0.89. The correlation of the ICP-AES results with respect to those of the ISEs attached to either electronic measurement system demonstrate that the ISEs together with the reader can provide reliable measurements of soil potassium content for field-based agricultural diagnostics.

Using the bill of materials (BOM) for the combined ISE strips and reader (Tables SI-1, SI-2) we calculated that single-use individual strips cost 0.26 USD each and that the reusable reader costs 8.63 USD. These BOM calculations include material costs for volumes of 5,000–10,000 units, but not additional costs such as labor, manufacturing, packaging, distribution, and marketing. While these costs may significantly increase the final retail price of the product, we expect that they will be offset by advances in manufacturing throughput and lower bulk prices to result in a final price which is similar to that estimated from our BOM calculations. This price is consistent with the desired cost constraints expressed during our conversations with potential users in rural India, offering the possibility of inexpensive point-of-use soil diagnostics in this and similar resource-limited environments.

## Conclusion

We presented an integrated electrode-reader system for in-field measurement of soil ion chemistry, which is critical for soil health optimization. Using screen-printing and drop-casting of commercially available materials, we fabricated ISEs that exhibited consistent potentiometric sensitivities and limits of detection for potassium determination, as well as high selectivity with limited interference from other competing ions. Strontium chloride extraction solution enabled measurement of soil potassium concentrations while only decreasing the limit of detection of the ISE by one order of magnitude, outperforming the Kelowna, Morgan, and Modified Morgan extractant solutions. We also developed a potentiometric reader, which together with the ISEs and strontium chloride soil analyte extraction protocol demonstrated consistent results with potassium content as measured via ICP-AES. Ongoing work is dedicated to developing a full macronutrient test strip, with parallelized channels for nitrogen, phosphorus, and potassium measurement, as well as assessment of sensor storage, lifetime, and reliability in the field. Once a reliable and complete macronutrient strip is fully realized, a micronutrient strip which can test for analytes such as boron, zinc, calcium, and Iron could also be developed, as ionophores for these ions are commercially available. This technology, paired with an informatic mobile phone based fertilizer recommendation system, could ultimately enable low cost, point-of-use soil testing and nutrient management for rural farmers worldwide.

## Supporting information

S1 Fig Circuit diagram for handheld reader. Further description of the configuration, component selection, and software programming for the reader is provided in S1 Text, and a detailed procedure for measuring a soil sample with the reader is provided in S1 Protocol.
(TIF)

S2 Fig Accurate bin prediction error as a function of standard error in system. A model for determining accurate bin prediction rates was created using MATLAB. Briefly, a normal distribution of soil potassium levels were assumed, and then the range was split into five “bins” as is typical for many soil testing labs: “Low”, “Low-Medium”, “Medium”, “High”, and “Excessive”. The final model was able to calculate how standard errors in potential across the system mapped to predictive errors for the final potassium level. Using this model, we were able to generate a benchmark that total standard error should remain smaller than ±5.4mV so as to maintain a commensurate 80% predictive accuracy rate. For the reader, there are two principle sources of error: the differential module and quantization resulting from the analog to digital converter. Noise will be effectively minimized by removing outliers and averaging a large number of samples. Resistors with 1% tolerance and equal values were used for the differential module. This results in a total tolerance of 3%, or 6mV for an input difference of 200mV. Typical error will be lower. The ADC gives values over a range of 1024 steps, ranging from 0 V to 2.56 V using internal reference in the micro-controller. This results in a maximum possible error of 2.5 mV, and a typical error of 1.25 mV. The actual voltage will always be larger than the reported voltage by up to these levels.
(TIF)

S3 Fig SolidWorks model of the PCB and case design. The case of the reader was designed in SolidWorks, using the board design from Eagle as a guideline. The case is comprised of two shells, each 3D printed on the Ultimaker+ using default settings and PLA filament (Ultimaker). The shapes of the shells were roughly rectangular, with slight 10 degree depressions on the sides to afford gripping. The shells were adorned with cut extrusions which delineated where PCB components (i.e. buttons, LEDs, temperature sensor, on/off switch) extend outside. Rectangular holes are found on the bottom of the case to accommodate mating with the peg protrusions on the walls of the receptacle, which is used for containing the solutions for soil testing. The two shells were connected together via a lip/groove formulation and some low-tack Dot Shot Pro adhesive (Staples). M4 screws (McMaster) held together the PCB against the back case along with the aid of threaded brass plastic inserts (McMaster) which press-fit into boss extrusions in the back for extra stiffness. Finally, the battery of the PCB fit into a walled extrusion in the backside of the PCB, which ensured the battery did not disconnect from the power cables and did not move during operation.
(TIF)

S1 Text Description of reader configuration, component selection, and software programming. (PDF)

S1 Protocol Procedure for measuring a soil sample with the reader and receptacle. (PDF)
