# Supplementary figures and images for: In-field determination of soil ion content using a handheld device and screen-printed solid-state ion-selective electrodes
Source: PLoS One. 2018 Sep 25;13(9):e0203862. doi: 10.1371/journal.pone.0203862 (PMC6155443; doi:10.1371/journal.pone.0203862)

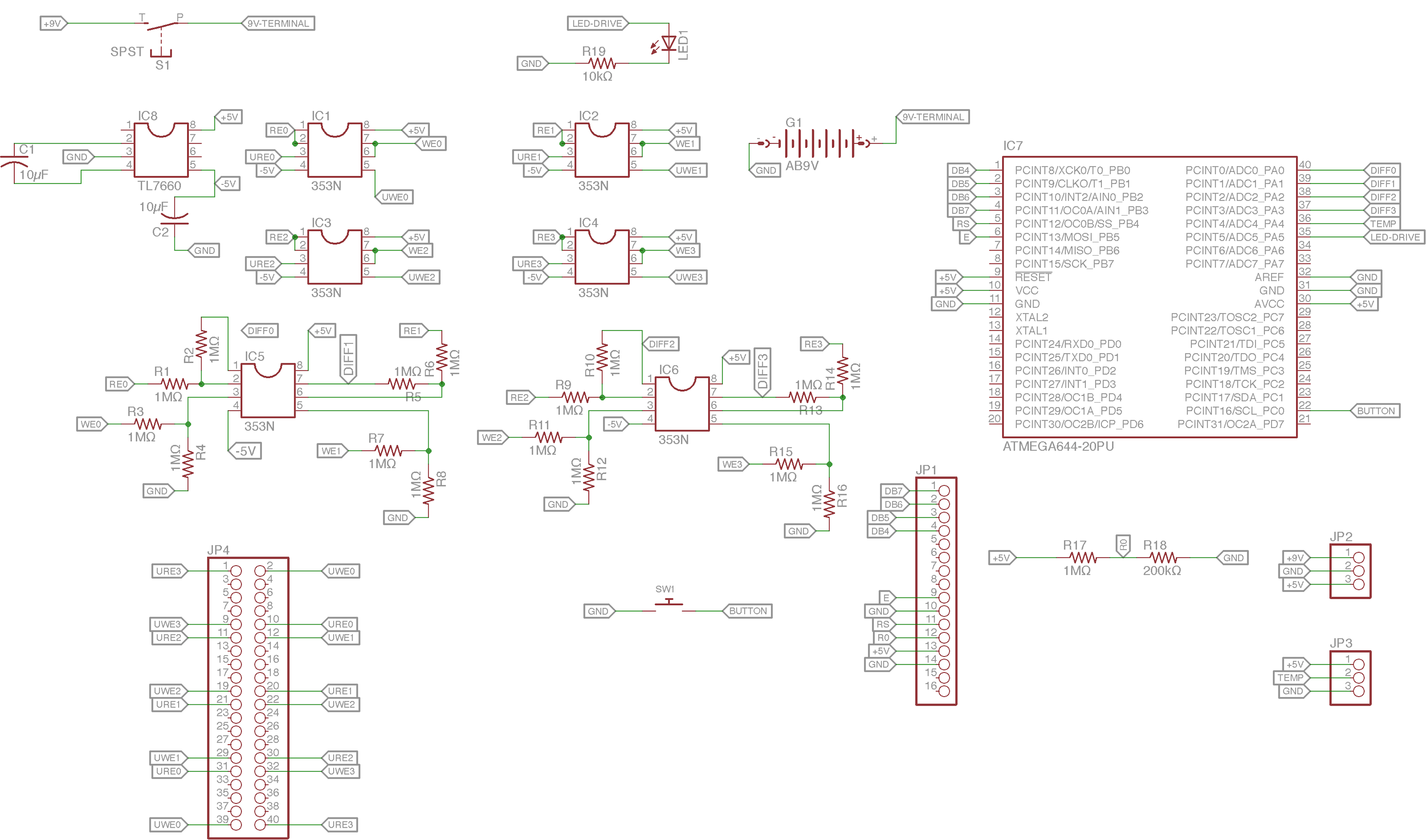

Supplement: S1 Fig — Further description of the configuration, component selection, and software programming for the reader is provided in S1 Text, and a detailed procedure for measuring a soil sample with the reader is provided in S1 Protocol. (TIF) [file pone.0203862.s001.tif]

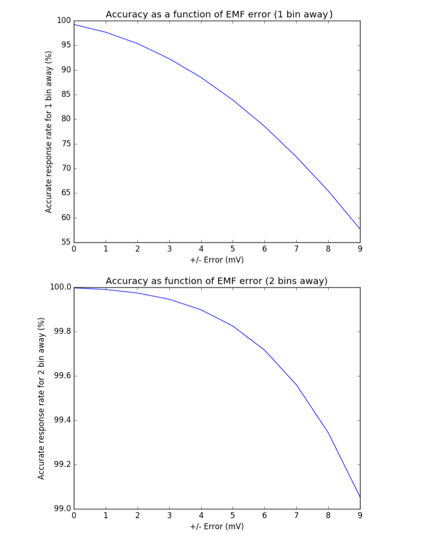

Supplement: S2 Fig — A model for determining accurate bin prediction rates was created using MATLAB. Briefly, a normal distribution of soil potassium levels were assumed, and then the range was split into five “bins” as is typical for many soil testing labs: “Low”, “Low-Medium”, “Medium”, “High”, and “Excessive”. The final model was able to calculate how standard errors in potential across the system mapped to predictive errors for the final potassium level. Using this model, we were able to generate a benchmark that total standard error should remain smaller than ±5.4mV so as to maintain a commensurate 80% predictive accuracy rate. For the reader, there are two principle sources of error: the differential module and quantization resulting from the analog to digital converter. Noise will be effectively minimized by removing outliers and averaging a large number of samples. Resistors with 1% tolerance and equal values were used for the differential module. This results in a total tolerance of 3%, or 6mV for an input difference of 200mV. Typical error will be lower. The ADC gives values over a range of 1024 steps, ranging from 0 V to 2.56 V using internal reference in the micro-controller. This results in a maximum possible error of 2.5 mV, and a typical error of 1.25 mV. The actual voltage will always be larger than the reported voltage by up to these levels. (TIF) [file pone.0203862.s002.tif]

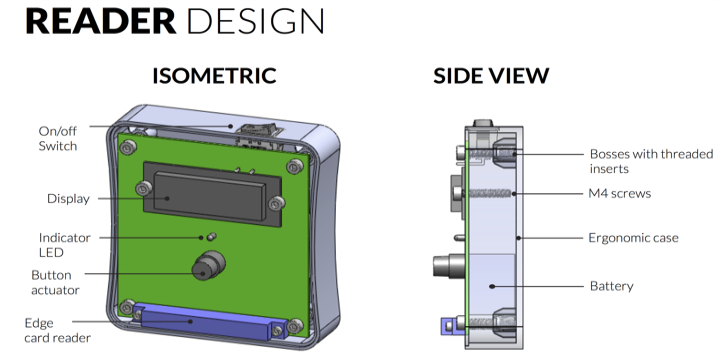

Supplement: S3 Fig — The case of the reader was designed in SolidWorks, using the board design from Eagle as a guideline. The case is comprised of two shells, each 3D printed on the Ultimaker+ using default settings and PLA filament (Ultimaker). The shapes of the shells were roughly rectangular, with slight 10 degree depressions on the sides to afford gripping. The shells were adorned with cut extrusions which delineated where PCB components (i.e. buttons, LEDs, temperature sensor, on/off switch) extend outside. Rectangular holes are found on the bottom of the case to accommodate mating with the peg protrusions on the walls of the receptacle, which is used for containing the solutions for soil testing. The two shells were connected together via a lip/groove formulation and some low-tack Dot Shot Pro adhesive (Staples). M4 screws (McMaster) held together the PCB against the back case along with the aid of threaded brass plastic inserts (McMaster) which press-fit into boss extrusions in the back for extra stiffness. Finally, the battery of the PCB fit into a walled extrusion in the backside of the PCB, which ensured the battery did not disconnect from the power cables and did not move during operation. (TIF) [file pone.0203862.s003.tif]
