# Supplementary material for: In-field determination of soil ion content using a handheld device and screen-printed solid-state ion-selective electrodes
Source: PLoS One. 2018 Sep 25;13(9):e0203862. doi: 10.1371/journal.pone.0203862 (PMC6155443; doi:10.1371/journal.pone.0203862)
Supplement: S1 Text — (PDF) [file pone.0203862.s004.pdf]

Description of Reader Configuration, Component Selection, and Software Programming

Reader Configuration

The reader consists of four ISEs, each consisting of a working and reference electrode pair, yielding measurements which can be averaged for greater accuracy. Two operational amplifier (op-amp) stages were used to measure the potential difference at each ISE. The initial stage of the circuit for each ISE is a buffer module at the working and reference electrodes to allow for the measurement of voltage while minimizing current flow between the solution and the reader edge card (EBC20DRAS). LF353-N dual chip JFET op-amps were selected due to their low input bias current as described in the Component Selection section. In the second stage, a differential op-amp circuit output the potential difference between the working and reference electrodes for conversion at the ADC pins on the micro-controller. No additional offset voltage was required for potassium measurement, as the measured potentials were always positive. Moreover, no hardware filtering was necessary, with the unfiltered amplified output sent to an ADC on the microcontroller (ATMEGA328P) for further processing.

In addition, another ADC on the microcontroller converts the input from a simple thermistor (MCP9700), allowing calculations to account for the ambient temperature at the exposed thermistor header. The microcontroller handled user interactions and calculated the potassium concentrations via the differential op-amp modules, thermistor, and a single push button for user input. Output is given from the micro-controller to an LED light with a current-limiting 10-kΩ resistor going to ground. The LED flashes when the device is measuring and is left on when a task must be performed or results are ready. The microcontroller also outputs to an LCD screen (LCM-S01602DTR/M) which can report to the users the solution that needs to be measured, the time remaining, and the resulting concentration. A Hitachi HD44780 controller is built into the LCD screen, limiting the number of pins required and greatly simplifying the software and hardware.

As several components, including the op-amps, microcontroller, thermistor, and LCD, require a supply voltage of 5 V, a buck converter (L7805CV) was used to convert the voltage supplied by a 9-V battery to a constant output of 5 V regardless of variation in the battery input voltage. Negative voltage required by the op-amps was supplied by a CMOS DC-DC voltage converter (TL7660) configured to provide -5V to the V- rail, with an input to the V+ rail of +5V.

Component Selection

The op-amp selected was the LF353-N, a dual chip JFET op-amp with an internally compensated offset voltage. The JFET input device provides wide bandwidth, low input bias currents and offset currents. What would be a negligible level of current in other applications could in this instance cause a significant change in voltage level as a result of using open-circuit potentiometry. By experimentation, a general purpose op-amp, such as a 741, allows enough current to pass through in order to change the concentration of ions being measured, so that over the relatively short period of time needed to measure the voltage potential would change this value. It was therefore determined that the standard internal input impedance of general purpose op amps, 10^6 Ω, was insufficient for our purpose as it resulted in a typical input bias current of 80 nA, and a maximum of 200 nA at 25 °C. However, op-amps with a higher level of input impedance (10^{12} Ω) were sufficient for these purposes, resulting in a typical input bias current of 50pA, which was found to be low enough for our purposes. The 353 also has an internally trimmed offset voltage to reduce error, and is very low cost.

The ATMEGA 644 micro-controller was used because of its familiar Arduino programming environment, easy setup, and wide range of features. It has an internal oscillator, saving the additional hardware of an external crystal oscillator and appropriate capacitors. It also has seven analog-to-digital converters and an additional three banks of eight general-purpose digital input/output pins, providing adequate scope for the entire project. It also provided more than enough programming memory for our project.

The buck converter used was a L7805CV, since it was low cost and easy to implement. It had a output voltage tolerance of 2% and high temperature range.

The TL7660 was used for the voltage converter, as it had several features. It is simple to implement, requiring only two external capacitors that do not require a high level of accuracy. No external diode is needed. It has a typical power efficiency of 98% and a 99.9% voltage conversion efficiency, providing more than adequate accuracy for our purposes.

The thermistor used was MCP9700, as it was easy to implement and provided necessary range and accuracy for our purposes.

The LCD used was the LCM-S01602DTR/M, as it had an internal Hitachi HD44780 controller and was relatively low cost. It has a display format of 16x2 characters.

The edge card reader used was an EBC20DRAS, bi-directional 40 pin (two rows of 20) female edge card connector that used right-angle output pins, which were necessary for the PCB so that the cards could be connected from the bottom while soaked in the solution being measured.

Software Programming

When the reader is turned on, the program begins immediately upon receiving power by initializing the general purpose IO ports, the LCD and the ADCs for future use. The number ’1’ is displayed in the middle of the LCD, representing the step that the user is on. When the button is pressed, a countdown of 60 seconds begins, which is displayed on the second line of the LCD. For the last 30 seconds, values are recorded from all four ADCs. When the countdown is proceeding, the LED begins flashing. It will remain solid when the user must perform an action or the result is ready. Step 2 and Step 3 continue in a similar manner, with Step 2 only lasting 10 seconds and recording no data as it is the wash step. Obvious outliers are then removed from the dataset, and averages are taken to determine the bin, ranging from the lowest bin "A" to excessive nutrients in bin "E". Cutoffs are hardcoded. For testing purposes, the button may be held down for 10 seconds to display the averages from each of the four ADC’s for both the soil solution and calibration solution steps. The average type and value are displayed one at a time, and may be scrolled through by pushing the button. At any point, the program may be restarted by turning off and on the rocker switch. No data will be saved in this instance.
