# Supplementary material for: In-field determination of soil ion content using a handheld device and screen-printed solid-state ion-selective electrodes
Source: PLoS One. 2018 Sep 25;13(9):e0203862. doi: 10.1371/journal.pone.0203862 (PMC6155443; doi:10.1371/journal.pone.0203862)
Supplement: S1 Protocol — (PDF) [file pone.0203862.s005.pdf]

Procedure for measuring a soil sample with the reader and receptacle

1. Aliquot 2.5 mL of soil using the soil scooper and add it to compartment 1 of the receptacle (demarcated with an etched "1" on the bottom).
2. Add 12.5 mL of solution 1 (extraction solution) by taking the beaker marked "1" and adding solution up to the fill line (demarcated with an etched line on the side of the receptacle).
3. Use the same spoon for aliquoting to mix the solution intermittently for 5 min to allow for full extraction of the ions.
4. Take an ISE strip and insert it into the bottom of the reader at the edge card port.
5. Turn on the reader by switching on the rocker switch at the top of the reader. A "1" will show at the center of the screen indicating the user should be performing steps relating to solution 1 and compartment 1 of the receptacle.
6. Place the reader with the strip into solution by mating the pegs at the top of the compartment 1 with the holes inside the case; this forms a stable connection for stable electrochemical measurement.
7. Press the button at the center of the reader to initialize the measurement; a countdown on the screen will occur for 60 seconds for which the last 30 seconds the microprocessor takes an average measurement across the four channels and saves each individual channel potential; at the end of this measurement a "2" will appear on the screen indicating it is time to move to compartment 2.
8. Add solution 2 (DI water) by taking the beaker marked "2" and adding solution to compartment 2 up to the fill line.
9. Place the reader with the strip into solution by mating the pegs at the top of the compartment 2 with the holes inside the case; this forms a stable connection for stable electrochemical measurement.
10. Press the button at the center of the reader to initialize the measurement; a countdown on the screen will occur for only 10 seconds; at the end of this measurement a "3" will appear on the screen indicating it is time to move to compartment 3.
11. Add solution 3 (calibration solution) by taking the beaker marked "3" and adding solution to compartment 3 up to the fill line.

12. Place the reader with the strip into solution by mating the pegs at the top of the compartment 3 with the holes inside the case; this forms a stable connection for stable electrochemical measurement.

13. Press the button at the center of the reader to initialize the measurement; a countdown on the screen will occur for 60 seconds for which the last 30 seconds the microprocessor takes an average measurement across the four channels and saves each individual channel potential; at the end of this measurement a letter ("A", "B", "C", "D", "E") will appear on the screen serving as the alpha-numeric result of the soil testing measurement.
